# Supplementary material for: Developments and applications of the OPTIMADE API for materials discovery, design, and data exchange
Source: Digit Discov. 2024 Apr 18;3(8):1509–33. doi: 10.1039/d4dd00039k (PMC11305395; doi:10.1039/d4dd00039k)
Supplement: DD-003-D4DD00039K-s001 [file DD-003-D4DD00039K-s001.pdf]

Electronic Supplementary Material (ESI) for Digital Discovery.
This journal is © The Royal Society of Chemistry 2024

Supplementary Information
Developments and applications of the OPTIMADE API for materials discovery, design, and data exchange

Matthew L. Evans,[1,2] Johan Bergsma,[3,†] Andrius Merkys,[4] Casper W. Andersen,[5] Oskar B. Andersson,[6] Daniel Beltrán,[7] Evgeny Blokhin,[8,9] Tara M. Boland,[10] Rubén Castañeda Balderas,[11] Kamal Choudhary,[12] Alberto Díaz Díaz,[11] Rodrigo Domínguez García,[11] Hagen Eckert,[13,14] Kristjan Eimre,[15] María Elena Fuentes Montero,[16] Adam M. Krajewski,[17] Jens Jørgen Mortensen,[10] José Manuel Nápoles Duarte,[16] Jacob Pietryga,[18] Ji Qi,[19] Felipe de Jesús Trejo Carrillo,[11] Antanas Vaitkus,[4] Jusong Yu,[15] Adam Zettel,[13,14] Pedro Baptista de Castro,[20] Johan Carlsson,[21] Tiago F. T. Cerqueira,[22] Simon Divilov,[13,14] Hamidreza Hajiyani,[21,‡] Felix Hanke,[23] Kevin Jose,[24] Corey Oses,[25] Janosh Riebesell,[24,26] Jonathan Schmidt,[27] Donald Winston,[28] Christen Xie,[19] Xiaoyu Yang,[29,30,31] Sara Bonella,[3] Silvana Botti,[32] Stefano Curtarolo,[13,14] Claudia Draxl,[33] Luis Edmundo Fuentes Cobas,[11] Adam Hospital,[7] Zi-Kui Liu,[17] Miguel A. L. Marques,[32] Nicola Marzari,[15,34] Andrew J. Morris,[35] Shyue Ping Ong,[19] Modesto Orozco,[7] Kristin A. Persson,[26,36] Kristian S. Thygesen,[10] Chris Wolverton,[18] Markus Scheidgen,[33] Cormac Toher,[37,14] Gareth J. Conduit,[24] Giovanni Pizzi,[15,34] Saulius Gražulis,[4,38] Gian-Marco Rignanese,[1,2,39] Rickard Armiento[6]

[1] *UCLouvain, Institut de la Matière Condensée et des Nanosciences (IMCN), Chemin des Étoiles 8, Louvain-la-Neuve 1348, Belgium*
[2] *Matgenix SRL, 185 Rue Armand Bury, 6534 Gozée, Belgium*
[3] *Centre Européen de Calcul Atomique et Moléculaire (CECAM), École Polytechnique Fédérale de Lausanne, Avenue de Forel 3, 1015 Lausanne, Switzerland*
[4] *Institute of Biotechnology, Life Sciences Center, Vilnius University, Saulėtekio av. 7, LT-10257 Vilnius, Lithuania*
[5] *SINTEF, P.O. Box 4760 Torgarden, NO-7465 Trondheim, Norway*
[6] *Materials Design and Informatics unit, Department of Physics, Chemistry and Biology, Linköping University, Sweden*
[7] *Institute for Research in Biomedicine (IRB Barcelona), Baldiri i Reixac 10-12, 08028 Barcelona, Spain*
[8] *Tilde Materials Informatics, Straßmannstraße 25, 10249, Berlin, Germany*
[9] *Materials Platform for Data Science, Sepapaja 6, 15551, Tallinn, Estonia*
[10] *Computational Atomic-Scale Materials Design, Technical University of Denmark, Kgs. Lyngby, Denmark*
[11] *Centro de Investigación en Materiales Avanzados, S.C. (CIMAV), Av. Miguel de Cervantes 120, Complejo Industrial Chihuahua, 31136, Chihuahua, Chih., México*
[12] *Material Measurement Laboratory, National Institute of Standards and Technology, Gaithersburg, MD, 20899, USA*
[13] *Department of Mechanical Engineering and Materials Science, Duke University, Durham, NC 27708, USA*
[14] *Center for Extreme Materials, Duke University, Durham, NC 27708, USA*
[15] *Theory and Simulation of Materials (THEOS), and National Centre for Computational Design and Discovery of Novel Materials (MARVEL), École Polytechnique Fédérale de Lausanne, 1015 Lausanne, Switzerland*
[16] *Universidad Autónoma de Chihuahua, Facultad de Ciencias Quimicas, 31125 Chihuahua, México*
[17] *Department of Materials Science and Engineering, The Pennsylvania State University, University Park, PA 16802, USA*
[18] *Department of Materials Science and Engineering, Northwestern University, Evanston, IL 60208, USA*
[19] *Department of NanoEngineering, University of California, San Diego, 9500 Gilman Drive, La Jolla, California, 92093-0448, United States*
[20] *National Institute for Materials Science, 1-2-1 Sengen, Tsukuba, Ibaraki, 305-0047 Japan*
[21] *Dassault Systèmes Germany GmbH, Am Kabellager 11-13, 51063 Cologne, Germany*
[22] *CFisUC, Department of Physics, University of Coimbra, Rua Larga, 3004-516 Coimbra, Portugal*
[23] *Dassault Systèmes, 22 Science Park, CB4 0FJ, United Kingdom.*
[24] *Theory of Condensed Matter, Cavendish Laboratory, Cambridge, United Kingdom*
[25] *Department of Materials Science and Engineering, Johns Hopkins University, Baltimore, MD 21218, USA*
[26] *Lawrence Berkeley National Lab, Berkeley, CA, USA*
[27] *Materials Theory, ETH Zürich, Wolfgang-Pauli-Strasse 27, 8093 Zurich, Switzerland*
[28] *Polyneme LLC, New York, NY 10038, USA*
[29] *Computer Network Information Center, Chinese Academy of Sciences, Beijing, 100083, China*
[30] *University of Chinese Academy of Sciences, Beijing, 101408, China*
[31] *Beijing MaiGao MatCloud Technology Co. Ltd., Beijing, 100149, China*
[32] *Research Center Future Energy Materials and Systems of the University Alliance Ruhr and Interdisciplinary Centre for Advanced Materials Simulation, Ruhr University Bochum, Universitätsstraße 150, D-44801 Bochum, Germany*
[33] *Humboldt-Universität zu Berlin, Institut für Physik and IRIS Adlershof, 12489 Berlin, Germany*
[34] *Laboratory for Materials Simulations (LMS), Paul Scherrer Institute (PSI), 5232 Villigen PSI, Switzerland*
[35] *School of Metallurgy and Materials, University of Birmingham, Edgbaston, Birmingham B15 2TT, United Kingdom*
[36] *Department of Materials Science and Engineering, UC Berkeley, Hearst Mining Memorial Building, Berkeley, 94720 CA, USA*
[37] *Department of Materials Science and Engineering and Department of Chemistry and Biochemistry, The University of Texas at Dallas, Richardson, TX 75080, USA*
[38] *Institute of Computer Science, Faculty of Mathematics and Informatics, Vilnius University, Naugarduko g. 24, LT-03225 Vilnius, Lithuania*
[39] *School of Materials Science and Engineering, Northwestern Polytechnical University, Xi'an, Shaanxi, 710072, China*
† Present address: Van 't Hoff Institute for Molecular Sciences, University of Amsterdam, PO Box 94157, 1090 GD Amsterdam, Netherlands
‡ Present address: Henkel, AID/Digital Twins & Data Analytics, 40589 Düsseldorf, Germany

PROVIDER	N_1	N_2	N_3	N_{tot}
AFLOW	704,302 (700,192)	63,017 (62,293)	413,797 (382,554)	3,530,330
Alexandria*	939,084	48,510	437,768	5,055,842
COD	458,249 (416,314)	4,082 (3,896)	34,739 (32,420)	512,282
CMR	2,811	386	0	16,789
JARVIS-DFT	9,017	1,426	8,084	77,096
Materials Cloud*	961,564	4,218	136,176	4,515,120
Materials Project	34,424 (27,309)	3,750 (3,545)	11,861 (10,501)	154,387
MPDD	811,136	80,195	490,900	3,975,666
MPOD	91	8	16	401
MPDS	-	-	-	507,178
NOMAD	4,451,056 (3,359,594)	587,923 (532,123)	2,092,989 (1,611,302)	12,116,021
odbx*	125,648 (55)	3,179 (54)	17,009 (0)	523,216
omdb	58,718 (58,718)	690 (690)	7,428 (7,428)	68,566
OQMD	261,400 (153,113)	15,375 (11,011)	81,673 (70,252)	1,226,781
TCOD	7,161 (2,631)	296 (296)	662 (660)	7,452
2DMatpedia	1,172	739	255	6,351

Table 1. The table from Ref.[1] recreated in March 2024, with new providers. The final column indicates the total number of structures served by each OPTIMADE API. Providers that serve multiple databases are indicated with ⋆. Results for Materials Cloud and Materials Cloud Archive have been aggregated under the same title. The corresponding values from the 2021 paper[1] are provided in brackets, where appropriate.

References

1 C. W. Andersen et al. OPTIMADE: an API for exchanging materials data. *Scientific Data*, 8:217, 2021. DOI: 10.1038/s41597-021-00974-z.
